# Improved Desulfurization Performance of Polyethyleneglycol Membrane by Incorporating Metal Organic Framework CuBTC

**DOI:** 10.3390/polym12020414

**Published:** 2020-02-11

**Authors:** Caibin Cai, Xiaotao Fan, Xiaolong Han, Jiding Li, Harsh Vardhan

**Affiliations:** 1 School of Chemical Engineering, Northwest University, Xi’an 710069, Shaanxi, China; caicb@nwu.edu.cn (C.C.); fanxt@nwu.edu.cn (X.F.); 2 State Key Laboratory of Fine Chemicals, Dalian University of Technology, Dalian 116024, China; 3 Department of Chemical Engineering, Tsinghua University, Beijing 100084, China; lijiding@mail.tsinghua.edu.cn; 4 Department of Chemistry, University of South Florida, 4202 E, Fowler Avenue, Tampa, FL 33620, USA; hvardhan@mail.usf.edu

**Keywords:** CuBTC nanoparticles, mixed matrix membranes, pervaporation desulfurization

## Abstract

In this paper, copper benzene-1,3,5-tricarboxylate (CuBTC) was incorporated into polyethylenglyol (PEG) to prepare a mixed matrix membrane (MMM) for pervaporation desulfurization. The characterization results showed that the prepared CuBTC particles had an ideal octahedral shape and micropores. The Cu^2+^ in CuBTC interacts with thiophene via π-complexation, thus enhancing the separation performance of the hybrid membranes. The effect of CuBTC content and the operating condition on the pervaporation performance of the MMMs was investigated. An optimal pervaporation separation performance was acquired with a permeation flux of 2.21 kg/(m^2^·h) and an enrichment factor of 8.79, which were increased by 100% and 39% compared with the pristine PEG membrane. Moreover, the CuBTC-filled PEG membrane showed a good stability in the long-term desulfurization under a high operating temperature of 75 °C for five days.

## 1. Introduction

Gasoline is one of the most prominent energy sources in society, as it can provide enough fuel for vehicles, ships and aviation aircraft. However, the presence of sulfur compounds pose a threat to the environment due to the fact that their combustion produces large quantities of harmful gas. Based on environmental requirements, the content of sulfur in gasoline must be limited. Accordingly, the reduction of the sulfur content of gasoline via hydrodesulfurization [1], adsorption desulfurization [2], oxidation desulfurization [3], alkylation desulfurization [4] and biocatalytic desulfurization [5] has been well reported. However, these desulfurization processes have drawbacks, so the latest high-efficiency deep desulfurization process needs to be expanded. 

Pervaporation (PV) is a separation technology with a low energy consumption, flexible operating conditions, and an environmentally friendly procedure that has been used to separate close-boiling or azeotropic liquid mixtures [6]. At present, common polymer membranes for pervaporation desulfurization include hydroxyethyl cellulose (HEC) [7], polyvinylpyrrolidone (PVP) [8], polydimethylsiloxane (PDMS) [9], polyethylene glycol (PEG) [10], and polyimide (PI) [11]. Owing to its favorable film-forming properties, flexibility, and good desulfurization performance, PEG is the most preferential polymer material for pervaporation desulfurization. 

To further strengthen their desulfurization performance, PEG membranes are modified by filling them with inorganic particles. For example, Lin et al. [12] put a copper Y (CuY) zeolite into a PEG membrane to produce CuY/PEG mixed matrix membranes (MMMs), and they studied the effects of different filling amounts, operating temperatures, and permeate side pressures on the membrane’s desulfurization performance. In our previous work, we introduced zeolitic imidazolate framework (ZIF-8) particles into PEG membranes to achieve an excellent desulfurization performance [13]. Many research works have shown that the transition metal ions of inorganic particles can interact with non-benzene aromatic compounds like thiophene via π-complexation to promote the separation performance of hybrid membranes [14]. CuBTC (copper benzene-1,3,5-tricarboxylate), as a metal framework material with distinct properties, has been incorporated into PDMS to enhance its desulfurization performance [15,16]. However, there has not yet been any report of incorporating CuBTC into PEG to improve its desulfurization performance. Hence, the motivation of this study was to put CuBTC into PEG to improve the desulfurization performance of the PEG membrane, as well as to study the influence of factors such as filling content, operating temperature, and feed concentration. It should be noted that CuBTC particles are synthesized by using a hydrothermal method that uses water and absolute ethanol as solvents. This method can ensure the uniformity of the prepared CuBTC particles, which is important for elevating the permeability and selectivity of the PEG polymer membrane. 

## 2. Experimental

### 2.1. Materials

PEG was purchased from Sigma-Aldrich (Saint louis, MO, USA), and it had an average molecular weight of 100,000. Both maleic anhydride and thiophene were provided by the Aladdin Industrial Corporation (Shanghai, China). *N*-heptane was acquired from Fuyu Fine Chemical (Tianjin, China). A trimethylamine solution (30 wt %) was received from Adamas Reagent Co., Ltd. Copper nitrate trihydrate (Cu(NO_3_)_2_·3H_2_O) was supplied by the Aladdin Industrial Corporation (Shanghai, China). Trimesic acid was also obtained from Aladdin Industrial (Shanghai, China). All above-mentioned reagents were invoked as procured without further purification. In addition, polyvinylidene fluoride (PVDF) porous membranes were prepared in our laboratory by adopting a non-solvent-induced phase separation method [17].

### 2.2. Membrane Preparation

#### 2.2.1. Synthesis of CuBTC Particles

The procedure of preparing the CuBTC particles adopted a hydrothermal synthesis method that was previously reported by Schlichte et al. [18,19]. Firstly, 0.42 g of trimesic acid was dissolved in a 24 mL equivoluminal mixture of deionized water and absolute ethanol. Then, this mixture was stirred for about 30 min, and 0.875 g of copper (II) nitrate trihydrate was also dissolved into this solution and stirred completely until a homogeneous solution was accomplished. After that, the mixture was transferred to a 50 mL Teflon-lined stainless steel autoclave, which was heated to 373.15 K for small crystals were yielded. At the next stage, the hydrothermal reactor was cooled down to ambient temperature, and the resulting crystals were filtered and washed by using a mixture of large amounts of deionized water and absolute ethanol. Finally, the crystals were activated at 393.15 K in a vacuum oven and placed in a vacuum dryer for additional steps.

#### 2.2.2. Preparation of CuBTC-filled PEG MMMs

**Figure 1:**
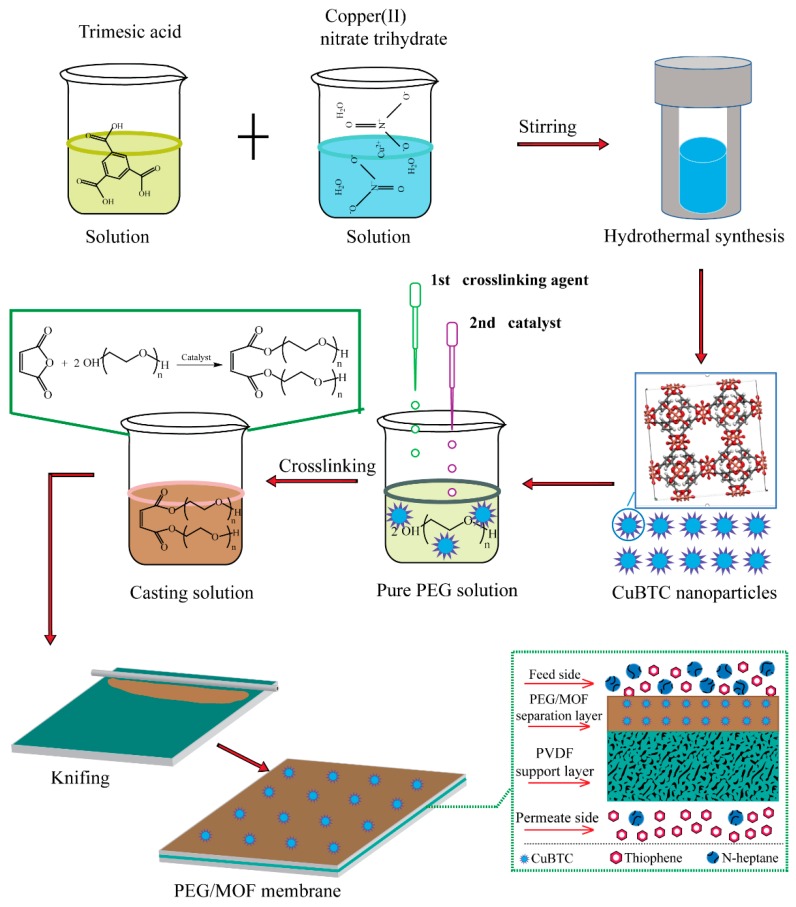
The schematic of preparation of the copper benzene-1,3,5-tricarboxylate (CuBTC)-filled polyethylenglyol (PEG) mixed matrix membranes (MMMs).

Firstly, the CuBTC particles were dispersed into a PEG aqueous solution and stirred evenly. Then, maleic anhydride, which acted as the crosslinking agent, and the trimethylamine solution, which served as the catalyst, were added to the solution. After thoroughly stirring with a magnetic stirrer, the mixed solution was cast on PVDF porous supporting membranes and allowed to stand at ambient temperature overnight to volatilize the solvents followed by crosslinking at a high temperature of 353 K for 5 h [6]. Subsequent to the crosslinking procedure was the following to maintain all membranes in a dry environment at room temperature. The mass ratio of CuBTC to PEG was 0%, 1%, 2%, 3%, 4% and 5%, which were deemed to be CuBTC-0%, CuBTC-1%, CuBTC-2%, CuBTC-3%, CuBTC-4%, CuBTC-5%. Figure 1 shows the preparation process of the mixed matrix membrane.

### 2.3. Characterization of CuBTC and Membranes

The functional groups of the CuBTC and the membranes were observed with Fourier transform infrared spectroscopy (FTIR, Bruker, Karlsruhe, Baden-Württemberg, Germany) ranging from 3500 to 500 cm^−1^ with a resolution of 4 cm^−1^. The surface and cross-sectional morphologies of the particles and membranes were analyzed with scanning electron microscopy (SEM, ZEISS, Jena, Thuringia, Germany). The crystal structures of the CuBTC particles and the CuBTC/PEG hybrid membranes were investigated by X-ray diffraction (XRD, Bruker, Karlsruhe, Baden-Württemberg, Germany) operating at 40 kV and 40 mA with a scan speed of 10°/min and a 2θ range of 5–50°; a Cu/Kα radiation source was used. The surface area and pore size distribution analysis test for the CuBTC particles was carried out with an automatic N_2_ adsorption–desorption device (BET, Quantachrome, Nova 4000, Boynton Beach, FL, USA).

### 2.4. Pervaporation Experiments

**Figure 2:**
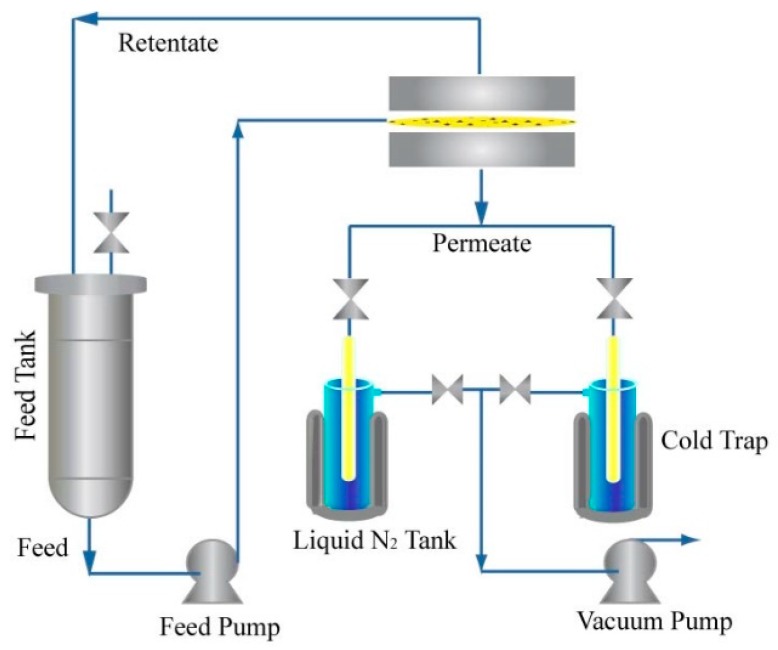
The schematic of the pervaporation apparatus.

The pervaporation equipment can be seen in Figure 2. The feed solution was the simulated gasoline, including thiophene and *n*-heptane, with a concentration ranging from 200 to 800 ppm. Analyses of the flux and permeability were performed by high-performance gas chromatography (GC9790, Zhejiang Fuli Analytical Apparatus, Hangzhou, China). The permeate flux is defined as follows:

(1) $$J=\frac{Q}{At}$$

where $Q\;\left(kg/\left(m^{2}\cdot h\right)\right)$ represents the total amount of permeate, $A\;\left(m^{2}\right)$ indicates the effective area of the membrane, and t (h) stands for operating time. The sulfur enrichment factor can be achieved in Equation (2).

(2) $$E=\frac{C_{P}}{C_{F}}$$

In Equation (2), E denotes the sulfur enrichment factor, and $C_{P}$ and $C_{F}$ denote the corresponding concentration of the component to be separated from the permeate and the feed, respectively. To more accurately evaluate the permeation driving force of the membrane itself, permeability and selectivity are introduced in Equations (3) and (4).

(3) $$P_{i}=J_{i}\frac{l}{P_{io}-P_{il}}=J_{i}\frac{l}{\gamma_{io}^{l}x_{io}^{l}P_{io}^{sat}-P_{il}}$$

(4) $$S=\frac{P_{i}}{P_{j}}$$

In Equation (3), the permeability is the membrane permeation flux after the normalization of the driving force, the membrane thickness, and the effective area of the membrane. $J_{i}\;\left(g/\left(m^{2}\cdot h\right)\right)$ is the component permeate flux; l (m) is the film thickness; $P_{io}^{sat}\;\left(Pa\right)$ is the pure component saturated vapor pressure; $P_{io\;}\left(Pa\right)$ and $P_{il}\;\left(Pa\right)$ are the partial pressure on the feed side and the permeate side, respectively, $\gamma_{io}^{l}$ is the activity coefficient of the component in the feed liquid; and $\chi_{io}^{l}$ is the mole fraction of the component in the feed. As for selectivity, it can be expressed as the ratio of the permeability of the thiophene to *n*-heptane in Equation (4):

## 3. Results and Discussions

### 3.1. Characterization of CuBTC Particles

**Figure 3:**
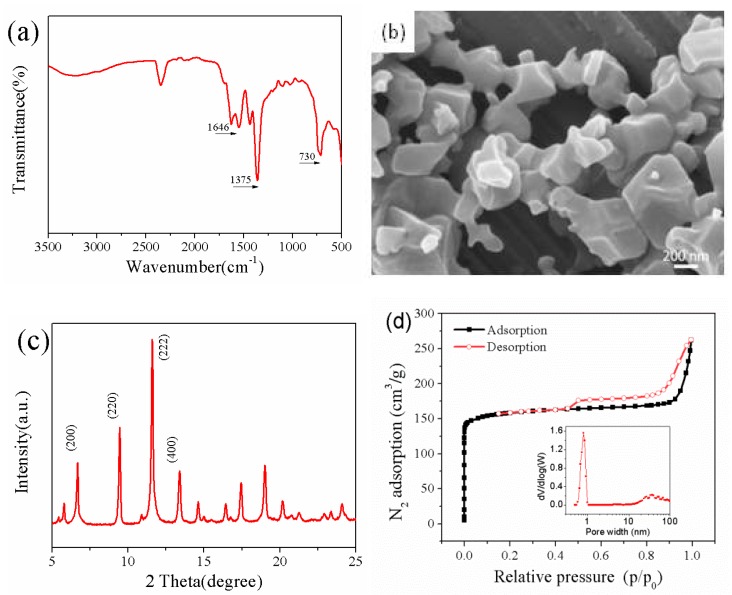
The FTIR (**a**), SEM (**b)**, XRD (**c**), and N_2_ sorption isotherms and pore size distribution (**d**) analyses of the CuBTC particles.

A series of methods such as FTIR, SEM and XRD were used to characterize the CuBTC particles. It can be seen from the FT-IR spectra of the CuBTC powder that is illustrated in Figure 3a that the strong characteristic bands of the CuBTC were located at 3398, 1646, 1375, and 730 cm^−1^ [15]. The peak at 3398 cm^−1^ could be attributed to the –OH groups that were contained in the water molecules of the crystal. The peak at 1646 cm^−1^ could be assigned to the stretching vibration of the C=O bond of the deprotonated trimesic acid. Additionally, the band at 1375 cm^−1^ corresponded to the stretching vibration of C=C, and the band at 730 cm^−1^ could be attributed to the scissoring vibration of the carboxylate ion. Figure 3b presents the SEM image of the CuBTC, which had a well-defined octahedral morphology [20]. Meanwhile, Figure 3c demonstrates that the CuBTC particles with characteristic diffraction peaks were consistent with the theoretical CuBTC in the XRD pattern [21]. As shown in Figure 3d, the BET surface area and pore size of the CuBTC particles are shown to be 623.88 m^2^/g and 0.968 nm, respectively. Owing to the presence of the amount of microporous structure inside the CuBTC particles, the N_2_ adsorption volume increased drastically at a low relative pressure. The subsequent near-horizontal platforms indicate that the micropores were fully filled without further adsorption. When reaching saturation pressure, the adsorption of agglomerates may have occurred in such a way that further increased the adsorption values. It should be mentioned that the adsorption curve and the desorption curve do not coincide, which could be credited to the existence of mesopores, and these phenomena agree with other literature [22].

### 3.2. Characterization of CuBTC/PEG Hybrid Membranes

#### 3.2.1. FT-IR Spectra of CuBTC/PEG Hybrid Membranes

**Figure 4:**
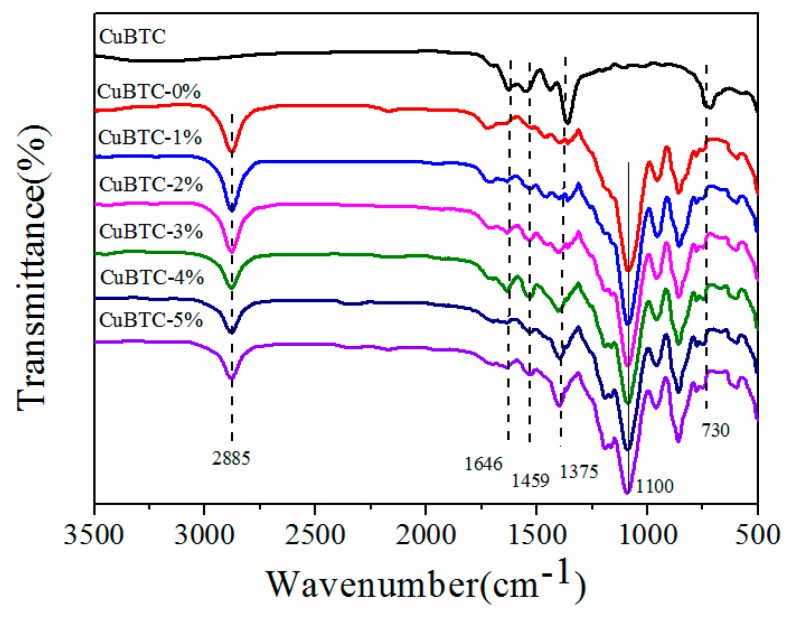
FT-IR spectra of the CuBTC, pristine PEG, and CuBTC-filled PEG MMMs.

The FT-IR spectra of the CuBTC/PEG mixed matrix membranes are demonstrated in Figure 4. It can be found that the peaks at 2885, 1459, and 1100 cm^−1^ were related to the deformation vibration of H–C–H, the asymmetric stretching vibration of H–C–H, and the axial vibration of C–O–C, respectively [6,23]. As mentioned above, the bands for the CuBTC powders were 1646, 1375, and 730 cm^−1^. The results show that the CuBTC particles were embedded in the PEG hybrid membranes for the reason that all the characteristic peaks of CuBTC could be found from the spectra of the hybrid membranes. Moreover, the intensity of the characteristic peaks was enhanced with the increase of the content of CuBTC.

#### 3.2.2. SEM Photographs of CuBTC-Filled PEG MMMs

**Figure 5:**
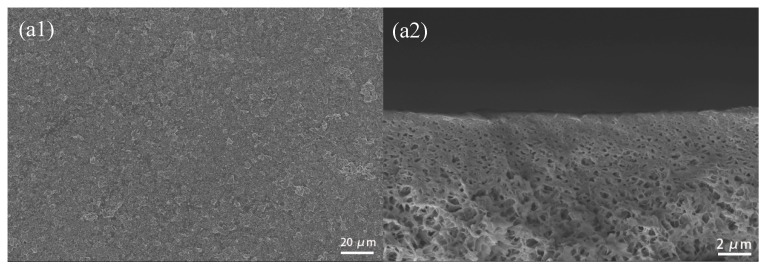

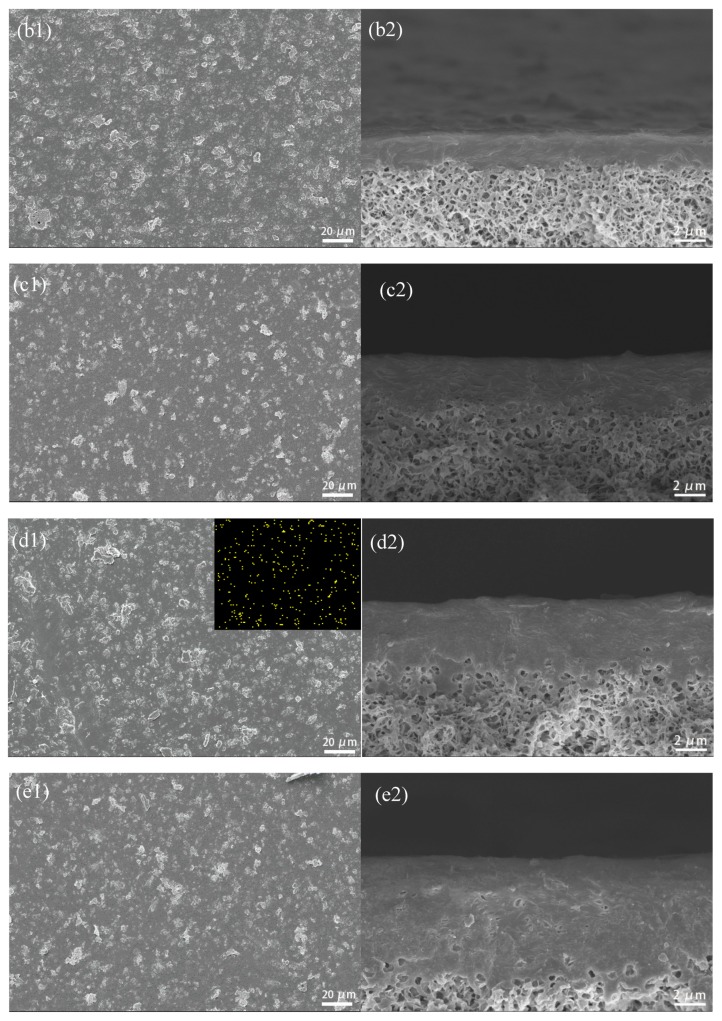

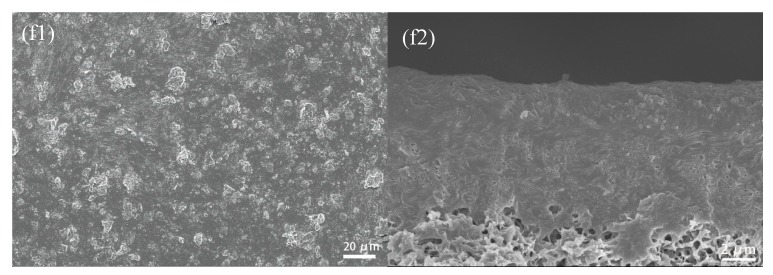
Surface and cross-sectional SEM images of the MMMs: (**a**) CuBTC-0%, (**b**) CuBTC-1%, (**c**) CuBTC-2%, (**d**) CuBTC-3%, (**e**) CuBTC-4% and (**f**) CuBTC-5%.

The surface and cross-sectional morphology of the CuBTC/PEG mixed matrix membranes are shown in Figure 5. By observing the membrane surficial images, it can be found that more and more CuBTC particles appeared on the surface of the membrane as the CuBTC content increased from 0% to 5%. To confirm the dispersal uniformity of the CuBTC particles, we performed an X-ray energy dispersive spectroscopy (EDS) analysis on CuBTC-3%. It could be seen that the Cu element was uniformly distributed on the surface. From the cross-sectional microstructure of the membranes, we could see that all the membranes consisted of dense separation layer and sponge-like support layer, which were tightly combined. As shown in Figure 5a2, the dense separation layer was very thin, and most of the PEG solution penetrated into the PVDF porous layer. Furthermore, the separation layer became thicker and thicker with the increase of the CuBTC loading from 1% to 5%, which may have caused a decrease in permeation flux.

#### 3.2.3. XRD Patterns of CuBTC-Filled PEG MMMs

**Figure 6:**
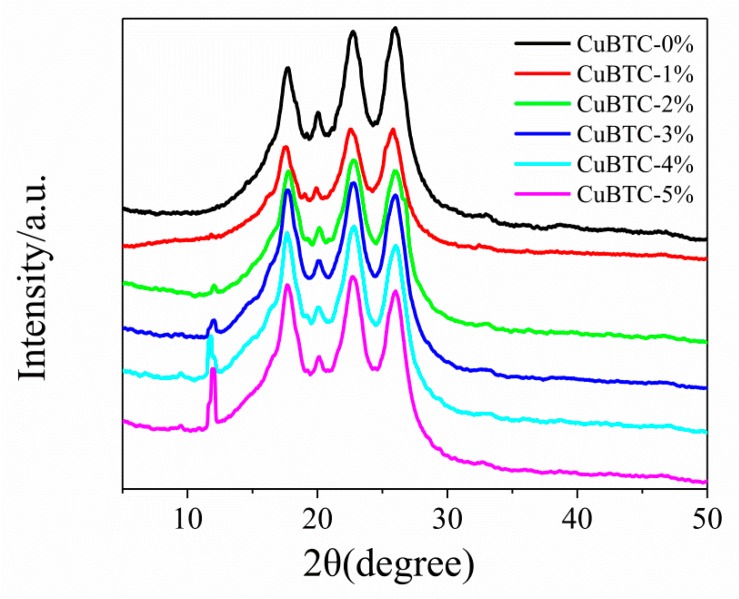
XRD patterns of the CuBTC-filled PEG MMMs.

Figure 6 confirms the XRD patterns of the CuBTC/PEG mixed matrix membranes. It was observed that the positions of the crystal diffraction peaks of PEG were 18°, 20°, 23° and 26°. The crystal diffraction peak of the CuBTC particles was around at 12°. Also, it can be seen that the position of the PEG diffraction peak and the peak’s shape and the peak’s intensity no change in the variety of CuBTC filling contents. However, there was a significant change trend for the intensity of the CuBTC diffraction peak. When the filling amount was 1%, the diffraction peak of CuBTC could not be clearly observed in the hybrid membrane. Meanwhile, the intensity of the diffraction peak grew stronger and stronger as the CuBTC content increased. All this information indicates that the crystallinity of the hybrid membrane could be enhanced with a high CuBTC content. Accordingly, the higher crystallinity meant a smaller free volume of the membrane [17], which may have resulted in a decrease of the permeation flux.

### 3.3. Pervaporation Performances of CuBTC-Filled PEG MMMs

#### 3.3.1. Effect of CuBTC Particle Content

**Figure 7:**
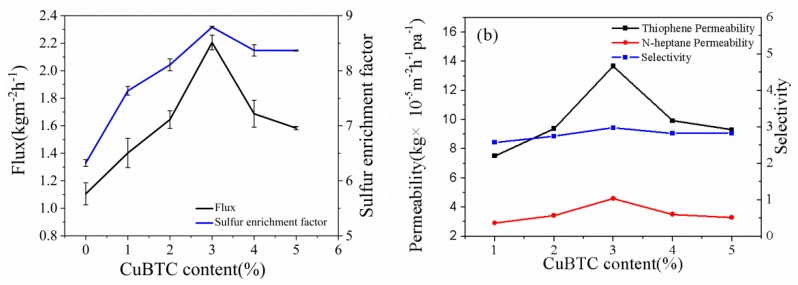
Effect of CuBTC content on (**a**) flux and sulfur enrichment factor; (**b**) permeability and selectivity of the CuBTC-filled PEG MMMs.

The effect of the CuBTC particle content on the membranes’ performance is shown in Figure 7. In the pervaporation experiment, the feed liquid temperature was 75 °C, the sulfur loading was 200 ppm, and the pressure of the permeation side was about 200 Pa. As the content of the CuBTC particles increased, both the flux and the enrichment factor experienced the same trend of firstly increasing and then decreasing. This result was consistent with the trend of permeability and selectivity of the membranes. The pore diameter of CuBTC was 0.96 nm, and the kinetic diameters of thiophene and *n*-heptane are only 0.46 nm [24] and 0.43 nm [25], respectively. When the CuBTC loading was less than 3%, the filled CuBTC provides molecular permeating channels to thiophene and *n*-heptane due to its larger pore size; thus, the permeation flux increased. The increase in the sulfur enrichment factor was probably due to the large number of active metal sites in the CuBTC particles, which could preferentially adsorb thiophene. An optimal pervaporation separation performance is achieved when the mass ratio of CuBTC to PEG was 3%, with a permeate flux of 2.21 kg/(m^2^·h) and an enrichment factor of 8.79, which were increased by 100% and 39%, respectively, when compared to the pristine PEG membrane. When the content of CuBTC was higher than 3%, the flux decreased. This could be ascribed to two reasons. First, the thickness of the hybrid membranes became thicker. Second, excessive particles in the membrane hindered the movement of the PEG molecular chain such that the free volume in the membrane was reduced, which was not conducive to component diffusion. As for the decrease of the sulfur enrichment factor, it can be explained as follows. The CuBTC particles’ distribution on the surface of the hybrid membrane with a high filling amount was significantly increased and partial particle accumulation occurrs, which may result in the defects between the PEG matrix and the CuBTC particles. Accordingly, the generated defects result in a decrease in the selectivity of the CuBTC-filled PEG membranes when the loading was higher than 3%. In view of the fact that the hybrid membrane with 3% CuBTC particle loading exhibited the highest sulfur enrichment factor and selectivity, the mixed matrix membrane that was prepared by this loading was selected for subsequent performance experiments.

#### 3.3.2. Effect of Feed Temperature

**Figure 8:**
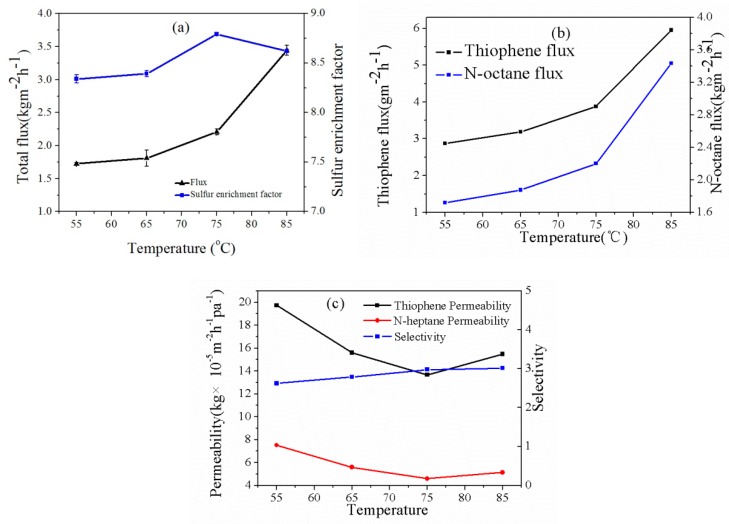
Effect of feed temperature on (**a**) total flux and sulfur enrichment factor; (**b**) thiophene flux and *n*-octane flux; and (**c**) permeability and selectivity of the CuBTC-filled PEG MMMs.

The effect of temperature on membrane desulfurization performance is illustrated in Figure 8. The experimental operating temperature range was from 55 to 85 °C. The feed sulfur content was about 200 ppm, and the pressure of the permeation side was about 200 Pa. As the temperature increased, the permeate flux increased gradually, while the sulfur enrichment factor tended to first increase and then decrease. As the operating temperature increased, the thermal motion of the PEG polymer chain could be enhanced, and larger molecular chain spacing increased the free volume fraction such that the membrane permeability was enhanced. In addition, enhanced vapor pressure on both sides of the CuBTC-filled PEG mixed matrix membrane which can promote the driving force for the mass transfer of the permeate component [6]. All these factors contributed to an increase in the permeate flux of the hybrid membranes, whereas the permeability of *n*-heptane and thiophene had the same trend (first decrease and then increase). The Arrhenius formula can be used to quantitatively describe the effect of operating temperature on membrane separation performance, as shown in the following equation.

(5) $$J_{i}=J_{oi}exp\left(\frac{-E_{P}}{RT}\right)$$

(6) $$\;lnJ_{i}=lnJ_{oi}-\frac{E_{P}}{RT}\;$$

In Equations (5) and (6), $J_{i}\;\left(kg/\left(m^{2}\cdot h\right)\right)$ is the split amount, $J_{oi}\;\left(kg/\left(m^{2}\cdot h\right)\right)$ is the permeation flux constant, $E_{P}\;\left(J\right)$ is the apparent activation energy of the permeation component, R (8.314 J/(mol·K)) is the gas molar constant, and T(K) is the operating temperature.

**Figure 9:**
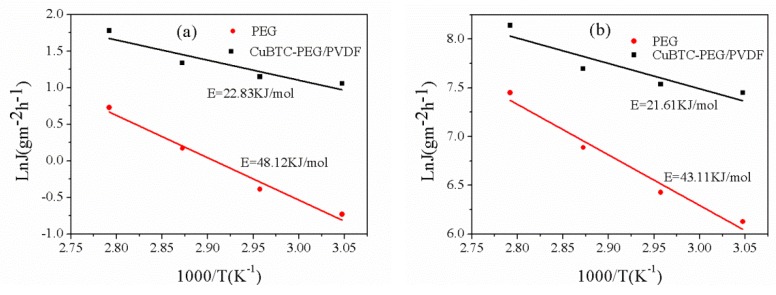
Arrhenius plots of the PEG and CuBTC-filled PEG MMMs for thiophene (**a**) and *n*-heptane (**b**).

Figure 9 shows the Arrhenius curve with a loading of 3% the CuBTC-filled PEG MMMs. It can be seen that the apparent activation energy of thiophene in the pure PEG membrane and the CuBTC-PEG/PVDF hybrid membrane was larger than that for *n*-heptane, which indicates that the thiophene flux was much more sensitive to the temperature during mass transfer. Therefore, as the operating temperature increased, the thiophene flux was increased to be larger than that of *n*-heptane, which resulted in more thiophene molecules passing through the membrane to increase the membrane permeation flux [26]. The reason for the trend of the sulfur enrichment factor was that more thiophene and *n*-heptane were dissolved in the membrane material due to membrane swelling and the dominant solubility rate when the temperature was lower than 75 °C. Meanwhile, the diffusion rate of *n*-heptane in the channel provided by CuBTC was higher than that of thiophene, and more *n*-heptane passed through the mixed matrix membrane, thus leading to a decrease in sulfur enrichment factor when the temperature was higher than 75 °C. On the other hand, the selectivity and sulfur enrichment factor experienced the same trend as the temperature increased. It attributes to the decreasing selective adsorption ability of the CuBTC particles for thiophene at high temperatures, thus causing a decrease in the selectivity of the membrane.

#### 3.3.3. Effect of Feed Sulfur Content

**Figure 10:**
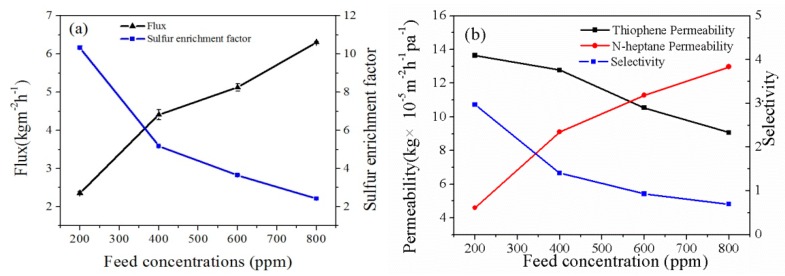
The effect of feed sulfur content on (**a**) total flux and sulfur enrichment factor; (**b**) permeability and selectivity of the CuBTC-filled PEG MMMs.

The effect of the feed sulfur content on the desulfurization performance of the CuBTC-filled PEG MMMs is demonstrated in Figure 10. The experimental temperature was 75 °C, and the CuBTC loading was 3%. When the feed sulfur content was increased, the permeate flux increased, whereas the sulfur enrichment factor decreased. Because the solubility parameter of PEG (δ = 20.1 MPa^1/2^) was similar to the solubility parameter of thiophene (δ = 20.15 MPa^1/2^). When the concentration of thiophene was much higher, the swelling degree of the membrane was also much higher, which led to the easier diffusion of the thiophene and *n*-heptane molecules, thus increasing the permeation flux. In addition, the permeability of *n*-heptane gradually increased, and the thiophene gradually decreased with an increase in the feed sulfur content, which resulted in a decrease of the the sulfur enrichment factor of the membrane.

#### 3.3.4. The Long-Term Stability of the Membranes

**Figure 11:**
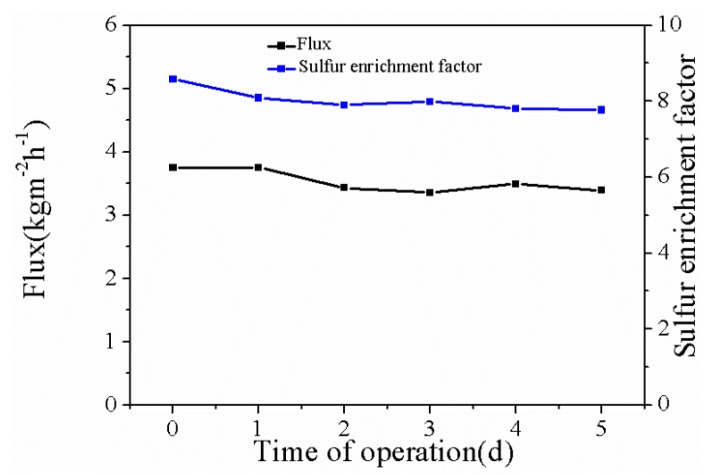
Long-term stability of the CuBTC-filled PEG MMMs.

Long-term stability is one of the important parameters of a membrane. The experimental temperature was 75 °C, and the CuBTC loading was 3%. Figure 11 shows that both the flux and sulfur enrichment factor were almost steady. Due to the binding force of the PEG-activated layer and the PVDF substrate, the good combination between the CuBTC particles and the PEG matrix, and the excellent dispersal uniformity of the CuBTC particles, the separation performance of the CuBTC-filled PEG MMMs exhibited excellent stability.

#### 3.3.5. Comparison of Pervaporation Performance

Table 1 illustrates the desulfurization performance of different membranes. Compared to the pristine PEG membrane, the incorporation of metal organic framework (MOF) particles can promote the sulfur enrichment factor of membranes. The sulfur enrichment factor of the prepared CuBTC-filled PEG membrane in our present work was higher than that of other CuBTC-filled polymer membranes.

## 4. Conclusions

CuBTC particles were prepared through hydrothermal synthesis and were integrated into a PEG matrix to generate mixed matrix membranes for gasoline desulfurization. SEM patterns and XRD results revealed that CuBTC particles were successfully put in the PEG membrane and exhibited a good compatibility. The pervaporation desulfurization performance of the membrane was comprehensively evaluated under different filling amounts, operating temperatures, and feed concentrations. An optimal pervaporation separation performance was achieved when there was a 3wt% CuBTC loading with a permeation flux of 2.21 kg/(m^2^·h) and an enrichment factor of 8.37, which were increased by 100% and 39%, respectively, when compared to the pristine PEG membrane. This study proves that CuBTC is a kind of ideal MOF material for improving the desulfurization performance of PEG membranes.

## Figures and Tables

**Table 1:** Desulfurization performance of different membranes.

Membrane	Feed	C (μg/g)	T (°C)	Flux (kg/(m^2^·h)	Enrichment Factor	Reference
PEG-PES	FCC gasoline	900	100	3.37	3.63	[27]
PEG-PU	FCC gasoline	1200	110	2.5	4.03	[28]
PEG-CuY	FCC gasoline	1190	110	3.19	2.95	[12]
CuBTC-PeBAX	Thiophene/octane	1300	70	16.45	4.04	[15]
CuBTC-PDMS	Thiophene/*n*-octane	1300	40	5.24	5.20	[16]
CPO-27-Ni--PDMS	Thiophene/*n*-heptane	200	45	5.92	4.05	[26]
ZIF-8-PEG	Thiophene/*n*-heptane	200	75	1.96	8.93	[6]
CuBTC-PEG	Thiophene/*n*-heptane	200	75	2.206	8.37	This work
